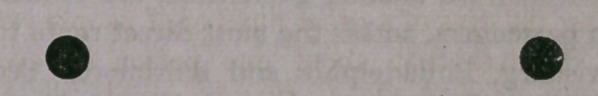# The Blind Spot

**Published:** 1871-01

**Authors:** 


### "THE BLIND SPOT."

It is perhaps, not generally known that every one has a blind spot in his eye,—a place where no vision is performed. The image of every object upon which your eyes are directed, is formed upon the retina, a nervous expansion of the optic nerve, which corresponds to the collodion used by the photographer in taking pictures. At a point on this retina, where the optic nerve comes through the several coatings of the eye, to spread itself over the inside of the eye for the reception of the image, is a perfectly blind spot. And, if you turn your eye in such a manner as to let the image of the object fall upon this particular spot, you will be unable to see the object. To convince you of this fact, we will ask you to close your right eye, and look steadily at the spot to your right, as here represented:

Hold the journal from twelve to fifteen inches from your face, keeping your eye steadily affixed upon the dot; bring the paper slowly nearer your eye, when you will find at a certain distance—about six or seven inches from the eye —that the *left* spot will entirely disappear; but, as you bring the paper nearer to you, or carry it farther off, it will become instantly visible again.

It has been found experimentally, that the reason of this blindness, is the image in the example given, falls directly upon the spot where the optic nerve enters the eye, and this point being "blind," no image is formed there. The optic nerve is considered by most people, to be the seat of vision, whereas it is only used in transmitting a knowledge of what has been photographed upon the retina, to the brain, where the mind takes cognizance of it, and we are said to see.

A HARD STORY.—A physician in Illinois tells a tough story, but doubtless a true one, to the effect that a patient of his, sixty years of age, in a street fight twelve years ago, had a knife thrust into his back, when a portion of it was broken off and remained in his person. A few weeks ago, in a violent paroxysm of coughing, he threw from his lungs, an ounce or two of pus, in which was found the knife-blade, an inch long, much corroded, and which had been lodged there twelve years before.

## Figures and Tables

**Figure f1:**